# Algae-Based Biopolymers for Batteries and Biofuel Applications in Comparison with Bacterial Biopolymers—A Review

**DOI:** 10.3390/polym16050610

**Published:** 2024-02-23

**Authors:** Jnanada Shrikant Joshi, Sarah Vanessa Langwald, Andrea Ehrmann, Lilia Sabantina

**Affiliations:** 1 Faculty of Engineering Sciences and Mathematics, Bielefeld University of Applied Sciences and Arts, 33619 Bielefeld, Germany; jnanada_shrikant.joshi@hsbi.de (J.S.J.); sarah_vanessa.homburg@hsbi.de (S.V.L.); 2 Department of Apparel Engineering and Textile Processing, Berlin University of Applied Sciences—HTW Berlin, 12459 Berlin, Germany; lilia.sabantina@htw-berlin.de; 3 Department of Textile and Paper Engineering, Higher Polytechnic School of Alcoy, Polytechnic University of Valencia (UPV), 03801 Alcoy, Spain

**Keywords:** microalgal biofuel, algae-based biopolymers, bacterial biopolymers, polymer electrolyte, batteries

## Abstract

Algae-based biopolymers can be used in diverse energy-related applications, such as separators and polymer electrolytes in batteries and fuel cells and also as microalgal biofuel, which is regarded as a highly renewable energy source. For these purposes, different physical, thermochemical, and biochemical properties are necessary, which are discussed within this review, such as porosity, high temperature resistance, or good mechanical properties for batteries and high energy density and abundance of the base materials in case of biofuel, along with the environmental aspects of using algae-based biopolymers in these applications. On the other hand, bacterial biopolymers are also often used in batteries as bacterial cellulose separators or as biopolymer network binders, besides their potential use as polymer electrolytes. In addition, they are also regarded as potential sustainable biofuel producers and converters. This review aims at comparing biopolymers from both aforementioned sources for energy conversion and storage. Challenges regarding the production of algal biopolymers include low scalability and low cost-effectiveness, and for bacterial polymers, slow growth rates and non-optimal fermentation processes often cause challenges. On the other hand, environmental benefits in comparison with conventional polymers and the better biodegradability are large advantages of these biopolymers, which suggest further research to make their production more economical.

## 1. Introduction

Polymers are used in diverse parts of our lives. Nowadays, an increasingly important property of polymers is their sustainability. A polymer is regarded as sustainable if it is produced from waste, from biological renewable resources, or from recycled material, and if it can stay in the biological or technical cycle of a circular economy at the end of its life [1]. Usually, bio-based polymers are regarded more sustainable than oil-based ones, while there are differences in the global warming potential and other environmental concerns for different biopolymers [2].

**Figure 1:**
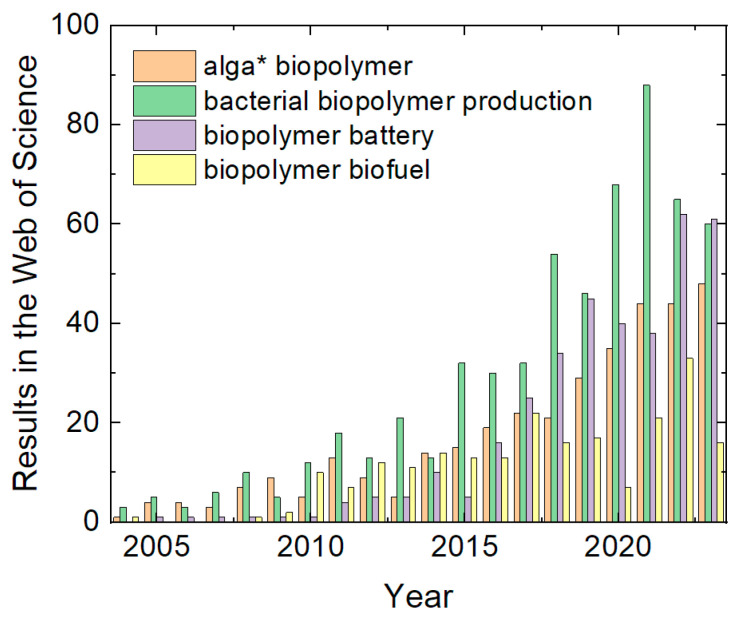
Results for different search phrases (with the asterisk as placeholder) in the Web of Science. Data collected on 25 December 2023.

The biopolymers which are often cited as especially sustainable alternatives to oil-based polymers are algal [3,4,5] and bacterial biopolymers [6,7,8], amongst others. This may be the reason why algal and bacterial biopolymers have been increasingly investigated during the last two decades, as depicted in Figure 1.

However, sustainability is not the only reason for the choice of a specific polymer. Their chemical and physical properties vary across a broad range; thus, each application necessitates specific polymers [9,10].

One of the current applications of polymers is their use in batteries [11]. In lithium–sulfur batteries, polymers can be used as electrodes, separators, binders. and electrolytes [12]. Similarly, in lithium-ion batteries, polymers are used as electrodes, separators, and electrolytes [13]. In lithium-polymer batteries, they serve as electrolytes [14]. In rechargeable batteries, electrochemically active polymers can be used, such as polyaniline, polypyrrole, different redox polymers, and others [15]. With new polymers with special physical properties, even completely new batteries can be developed, such as metal–polymer, organic, polymer–air, or all-polymer batteries [16,17]. Research on biopolymers for battery applications has also significantly increased in recent decades.

Another energy-related application of polymers can be found in the area of biofuels. Conductive polymers can be used for biofuel cells [18,19]. Biopolymers, such as polyhydroxyalkanoates (PHAs), can be used as a base for biofuel [20,21]. The research area of biopolymers for biofuel has also increasingly garnered interest in recent decades (Figure 1). 

This review provides a general overview of algal and bacterial biopolymers, before the specific applications of these biopolymers for battery applications and biofuels are discussed in detail. For this purpose, studies published during the last five years were taken into account.

## 2. Algal Biopolymers

### 2.1. Different Algal Biopolymers

Algae can produce a broad variety of biopolymers. Algae are diverse photosynthetic organisms that can be classified into various groups, including green algae, red algae, and brown algae. Algal biopolymers can be extracted from different parts of algae, such as the cell walls or intracellular compartments. These biopolymers possess unique properties that make them suitable for various applications in industries such as food, pharmaceuticals, biotechnology, and materials science. Microalgae can produce polyhydroxyalkanoates (PHAs), proteins, and polysaccharides [22]. 

**Figure 2:**
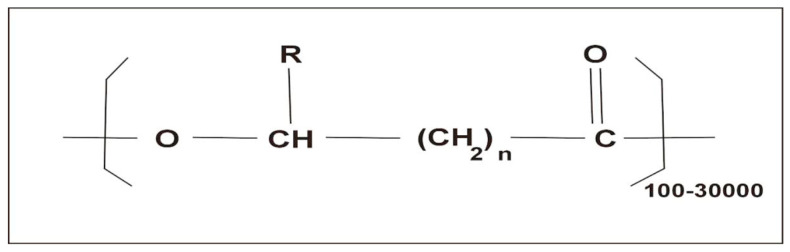
General structure of polyhydroxyalkanoates. Reprinted from [23] and copyright 2018, with permission from Elsevier.

PHAs consist of repeated ester units with a carbon chain that is connected to two oxygen atoms and an R-group (any group containing a carbon and a hydrogen atom that is attached to a molecule, refer Figure 2 [23]) and can be subdivided into short, medium, and long chain length PHAs [23]. Mechanically, they can have thermoplastic and elastomeric properties, are stable in air, and have low water solubility, but are easily dissolved in many other solvents [22]. They are often biocompatible and biodegradable and thus used in many biotechnological and biomedical applications [24,25].

Proteins consist of amino acids bonded by amide linkages, for building polypeptide chains [22]. Proteins are mainly used for food and thus to a lesser degree for bioplastic production. Investigations of algal proteins for bioplastic production were often carried out by blending the proteins as well as by comparing proteins received from different microalgae [26,27,28].

Cellulose is a biodegradable polysaccharide from D-glucose monomers with glycosidic bonds, but has other linkages and a rigid, elongated structure [29]. It can be found in algal cell walls [29] with different amounts and different crystallinities, depending on the algal species [30,31,32]. Cellulose is often used in composites to reduce its low thermal stability and high moisture absorption [29].

Starch is also a biodegradable polysaccharide from D-glucose monomers with glycosidic bonds, forming a helical structure [22]. Depending on the ratio of amylopectin to amylose, its mechanical properties vary [33]. Starch can be converted to monomers used in polymer synthesis, be a part of low-molecular-weight polymers, or be a filler in other polymers [34].

It should be mentioned that other biopolymers, such as poly(lactic acid) (PLA), can be produced from carbohydrates, which can be obtained from microalgae [35].

On the other hand, macroalgae, also called seaweed, can be used to produce the polysaccharides, i.e., alginic acid and carrageenan, from which bio-based plastics can be prepared [28]. Alginate is a linear copolymer of mannuronic acid and guluronic acid bonded by glycosidic linkages, with the varying blocks of these acids resulting in different physical and chemical properties [36]. While alginate is derived from brown algae, carrageenan is mostly extracted from red algae. This polysaccharide consists of D-galactose and 3,6-anhydro-D-galactose, similar to agarose, which is also derived from seaweed [37].

### 2.2. Algal Biopolymer Production

As mentioned before, the main reason to switch from well-known and well-suited oil-based polymers to biopolymers is the increased sustainability of the latter. Nevertheless, it is necessary to keep their whole life cycle in mind, regarding carbon footprint and the necessary energy to produce the required biopolymers. Algae biopolymers were shown to be advantageous in terms of biological composting and carbon sequestration as well as not using arable land [3]. One possibility to further reduce the ecological impact, which is often explored, is microalgae cultivation in wastewater, where wastewater treatment and biopolymer production are combined [38,39,40,41].

**Figure 3:**
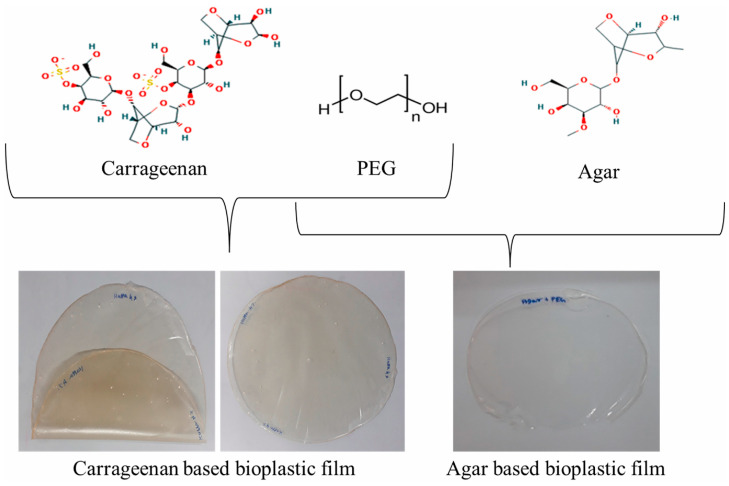
Bioplastic film developed using seaweed and polyethylene glycol (PEG) 3000 through the direct method. Reprinted from [43] and copyright 2024, with permission from Elsevier.

Some of the commonly used microalgae are *Nannochloropsis* sp., *Botryococcus braunii*, *Spirulina* sp., and *Chlorella* sp., while typical seaweeds from which biopolymers are obtained are *Ulva prolifera*, *Ecklonia radiate*, *Undaria pinnatifida*, etc. [42]. Common biopolymer production routes are the fermentation of algal biomass by microorganisms, biopolymer production inside the algal biomass, or blending algal biomass with additives [42]. Amongst these routes, the zero-waste concept would include directly converting algal biopolymers, while the conversion to monomers as bioplastic precursor offers a broader range of producible biopolymers [43]. Figure 3 shows algae-based biopolymer films using carrageenan and agar as examples [43].

**Figure 4:**
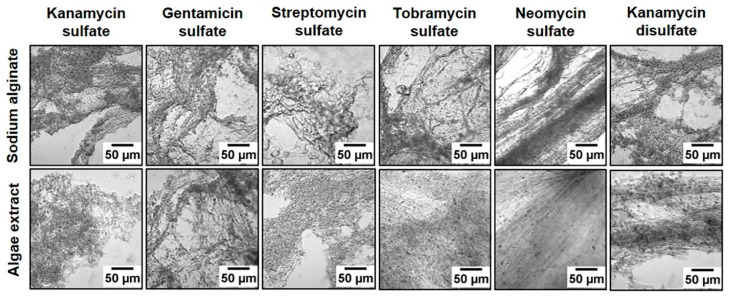
Texture of alginate polymers, showing that the addition of aminoglycoside to sodium alginate and algae extract resulted in more thread-like polymers for neomycin sulfate and kanamycin disulfate, whereas other aminoglycosides showed more granular polymers. From [46], originally published under a CC-BY license.

The large-scale commercial production of biopolymers can be performed in open or closed systems, with open systems having lower costs and larger production capacities but also higher contamination risks [44]. These non-standardized production methods, together with the broad range of algae used for biopolymer production and different production routes, reduces the reproducibility of the physical and chemical properties of the obtained biopolymers and thus should be optimized [45]. Besides extracting new polymers, this is why several researchers compared different biopolymers, obtained from various algal strands, and optimized algae cultivation and the subsequent biopolymer production [46,47,48,49], e.g., by adding chemicals to modify the texture of alginate polymers (Figure 4) [46].

### 2.3. Typical Applications of Algal Biopolymers

Algal biopolymers, especially from seaweed, are often used as food since they are abundant and sometimes especially healthy [50,51,52]. On the other hand, they can be used for food packaging due to their nontoxicity and biodegradability [53,54,55].

In addition, the specific physical and chemical properties of algal biopolymers make them suitable for different biomedical applications [56], such as nanocarriers for drug release in anticancer treatments [57] and wound dressings [58] or the well-known agarose gels for bacterial cultures and electrophoresis [56]. 

Other potential applications are related to fuel cells for wastewater cleaning [59] and energy harvesting and storage, as described in the next Sections. It should be mentioned that, depending on the planned application, the functionalization of the algal biopolymers is necessary, e.g., by crosslinking, by oxidation or substitution of functional groups, to reach the required physical and chemical properties [60]. On the other hand, specific processing techniques may support some applications, such as 3D printing, which has been investigated for algal polysaccharides by several research groups [61]. A more detailed description of some typical application areas is provided below.

#### 2.3.1. Applications of Algae-Based Biopolymers for Food

1. Algae-derived carrageenan in food and beverage industry

Carrageenan, a polysaccharide extracted from certain species of red algae, is extensively used as thickener and stabilizer in various food and beverage products. It enhances the texture, viscosity, and palatability of food products in dairy products, desserts, beverages, and also processed meats. Algae-derived carrageenan is natural, plant-based, and hence vegan-friendly, meeting the growing consumer demand for clean-label ingredients [62].

2. Algae-based agarose as a gelling agent

Agarose, a polysaccharide derived from seaweed red algae, is generally used as a gelling agent in food and beverage products. It forms stable gels even at considerably low concentrations, making it ideal for confectioneries, desserts, and microbiological culture media. Algae-derived agarose is vegetarian-friendly and allergen-free and possesses excellent gel strength and clarity, making it a strong alternative for synthetic or animal-derived alternatives [63].

3. Algae-based biopolymer films for food packaging

Algae-derived biopolymers, like alginate and ulvan, are currently researched for their potential application in natural and biodegradable food packaging materials. These biopolymer films are also flexible, and possess good barrier properties against moisture and oxygen, making them suitable for extending the shelf-life of perishable food products. Introducing algae-based biopolymers for food packaging meets the increasing demand for eco-friendly alternatives to reduce plastic waste and environmental pollution [64].

#### 2.3.2. Applications of Algae-Based Biopolymers in Pharmaceutics

1. Algal polysaccharides in drug delivery systems

Algal polysaccharides, like carrageenan and alginate, have been widely studied for their applications in drug delivery systems due to their advantageous properties of biocompatibility and biodegradability and also mucoadhesive properties. These polysaccharides can be formulated into different drug delivery systems, such as nanoparticles, hydrogels, and microparticles for controlled and targeted drug release [65].

2. Chitosan from algal sources in wound healing

Chitosan, a biopolymer derived from chitin, has been utilized in wound healing applications due to its antimicrobial properties, biocompatibility, and ability of promoting tissue regeneration. Chitosan derived from algae is advantageous due to its sustainable and renewable source compared to that of traditional crustacean-derived chitosan. Alginate–chitosan composite dressings have been developed for wound management, demonstrating enhanced healing properties [66].

3. Algal polysaccharides as anticancer agents

Algal polysaccharides, such as fucoidan and ulvan, are rapidly gaining attention for their potential anticancer properties. These biopolymers have demonstrated anti-proliferative, anti-metastatic, and immunomodulatory effects against various cancer cell lines. Fucoidan, in particular, has shown potential in inhibiting tumor growth and metastasis through multiple mechanisms, including the induction of apoptosis and the suppression of angiogenesis [67].

#### 2.3.3. Applications of Algae-Based Biopolymers in Biotechnology and Tissue Engineering

1. Alginate

Alginate, a polysaccharide extracted from brown algae, has been extensively applied in biotechnology and tissue engineering for its ability to form hydrogels and its biocompatibility. Alginate hydrogels are being employed as scaffolds for tissue engineering applications due to their ability to support cell growth and mimic the extracellular matrix (ECM) environment [68].

2. Carrageenan

Carrageenan, a polysaccharide derived from red algae, has shown promise as a scaffold material in tissue engineering. Carrageenan-based hydrogels are being employed for wound healing and drug delivery due to their biocompatibility and ability to form stable hydrogel networks [69].

3. Ulvan

Ulvan, a sulfated polysaccharide extracted from green algae, has gained attention in biotechnology and tissue engineering for its unique properties of biocompatibility, biodegradability, and ability to stimulate cell proliferation and tissue regeneration. Ulvan-based hydrogels have been investigated for applications such as controlled drug delivery and wound healing [70].

#### 2.3.4. Applications of Algae-Based Biopolymers in Cosmetics

1. Alginate

Alginate, a polysaccharide derived from brown seaweeds, such as *Laminaria* and *Macrocystis* species, is widely employed in cosmetics for its ability to form hydrogels and films, imparting moisturizing and emulsifying properties to skincare formulations. Alginate-based masks, creams, and serums are popular in the cosmetics industry due to their hydrating and soothing effects on the skin [71].

2. Carrageenan

Carrageenan, a sulfated polysaccharide extracted from red seaweeds, including *Kappaphycus* and *Eucheuma* species, is employed as a thickening and stabilizing agent in cosmetics such as various skincare and haircare products. Its film-forming properties contribute to the texture and viscosity of cosmetic formulations, enhancing their spreadability and shelf stability [72].

3. *Spirulina* extract

*Spirulina* is a blue-green microalga rich in proteins, vitamins, minerals, and antioxidants. Extracts from *Spirulina* are increasingly utilized in cosmetics for their anti-aging, antioxidant, and skin-nourishing properties. *Spirulina* extracts are incorporated into various skincare products, including facial masks, creams, and serums, to promote skin hydration, elasticity, and rejuvenation [73].

#### 2.3.5. Applications of Algae-Based Biopolymers in Biofuels

1. Algal lipids as feedstock for biofuel production

Algal lipids, particularly triglycerides, are a valuable feedstock for biofuel production due to their high lipid content and potential for conversion into biodiesel. Microalgae can accumulate lipids under specific growth conditions, making them a promising source for sustainable biofuel production [74].

2. Algal polysaccharides for bioethanol production

Polysaccharides extracted from algae, such as cellulose and starch-like compounds, can be enzymatically hydrolyzed into fermentable sugars, which can then be utilized for bioethanol production. Algal polysaccharides are advantageous due to the rapid growth, high carbohydrate content, and minimal land requirements of algae, making them promising feedstocks for sustainable bioethanol production [75].

3. Algae-based hydrocarbons for biofuel synthesis

Some algae species have the capability to produce hydrocarbons, such as alkanes and alkenes, which serve as precursors for renewable biofuels. These hydrocarbons can be extracted from algae biomass and can be further processed into drop-in biofuels with properties similar to petroleum-derived fuels [76].

#### 2.3.6. Environmental Applications of Algae-Based Biopolymers

1. Algal polysaccharides for heavy metal remediation

Algal polysaccharides, such as alginate and carrageenan, have been studied for their potential in heavy metal remediation from various water bodies. These biopolymers can form complexes with heavy metal ions, facilitating their removal through precipitation or adsorption processes. Research has demonstrated the effectiveness of algal polysaccharides in removing heavy metals like lead, cadmium, and copper from contaminated water, providing a sustainable and eco-friendly approach to water remediation [77].

2. Algal biopolymer-based membranes for wastewater treatment

Algal biopolymers have been investigated as membrane materials for wastewater treatment applications due to their biocompatibility, biodegradability, and low-cost fabrication. These membranes can effectively separate pollutants from water using ultrafiltration, nanofiltration, and reverse osmosis. Studies have shown that membranes fabricated from algal biopolymers exhibit high permeability, selectivity, and fouling resistance, making them attractive candidates for sustainable wastewater treatment systems [78].

3. Algae-based biopolymers for soil stabilization

Algae-based biopolymers have been examined for their potential in soil stabilization and erosion control applications. These biopolymers, when applied to soil surfaces, can form a protective layer that helps prevent soil erosion caused by wind and water. Research has demonstrated the effectiveness of algae-based biopolymers in improving soil structure, reducing sediment runoff, and enhancing vegetation growth in degraded ecosystems. This application offers a sustainable and environmentally friendly solution to soil erosion and land degradation issues [79].

#### 2.3.7. Applications of Algae-Based Biopolymers in Medical Devices

1. Alginate hydrogel

Alginate, derived from algae, has been widely used in the fabrication of hydrogels for various medical applications, including tissue engineering and drug delivery systems. Alginate hydrogels possess biocompatibility and tunable mechanical properties suitable for different tissue types [80].

2. Carrageenan-based wound dressings

Carrageenan, a polysaccharide extracted from red algae, has been applied in the development of wound dressings due to its biocompatibility, biodegradability, and ability to promote wound healing. Carrageenan-based dressings offer a moist environment for wound healing and can release therapeutic agents to aid wound recovery [81].

3. *Spirulina*-based scaffolds

*Spirulina*, a type of microalgae, has been employed in the fabrication of scaffolds for tissue engineering applications. *Spirulina*-based scaffolds offer benefits such as high porosity, biocompatibility, and the presence of bioactive compounds that can enhance cell proliferation and differentiation [82].

## 3. Bacterial Biopolymers

### 3.1. Different Bacterial Biopolymers and Their Production

**Figure 5:**
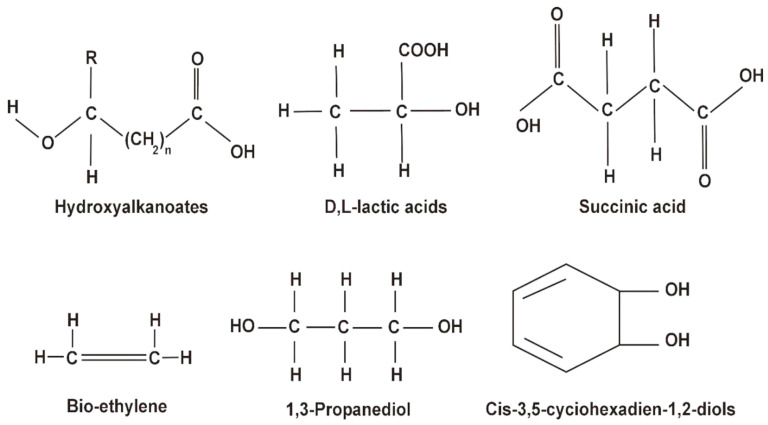
Molecular building blocks of bacterial biopolymers. Reprinted from [23] and copyright 2018, with permission from Elsevier.

Bacteria can produce diverse biopolymers using fermentation. There are many examples of polysaccharides such as dextran, xanthan gum, gellan gum, glucan, xanthan, pullulan, and glycogen [83]. Furthermore, bacterial biopolymers are also polypeptides such as polylysine, polyamides, polyesters, polyphosphates, and protein components [84,85]. The most common building blocks of bacterial biopolymers are depicted in Figure 5 [23].

Polysaccharides consist of sugars or sugar acids, which are either stored in bacterial cells or segregated to build a film on the cell surface or to become part of the biofilm, such as alginic acid or cellulose [86]. Dextran is a complex, branched glucan produced by special lactic acid bacteria, e.g., by the bacterium *Leuconostoc mesenteroides* [87], while xanthan gum is an anionic polysaccharide stemming from glucose/sucrose fermentation through the bacterium *Xanthomonas campestris* [88]. The anionic polysaccharide gellan gum is produced by *Sphingomonas elodea* [89].

**Figure 6:**
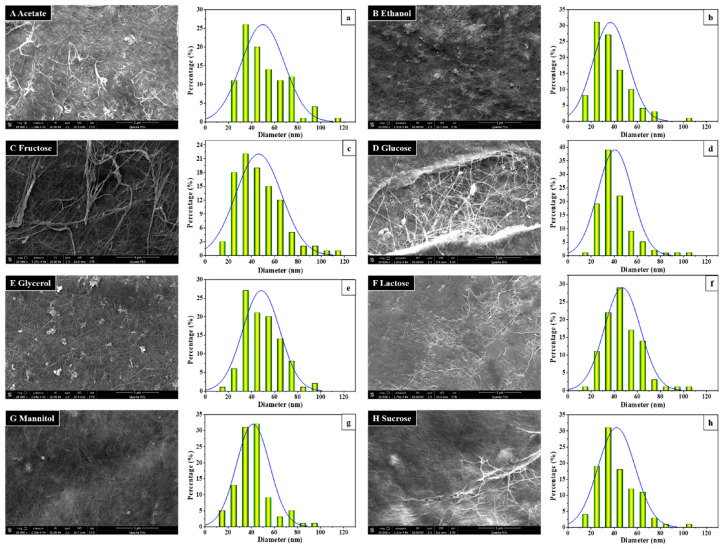
SEM images of the morphology (**A**–**H**) and diameter distribution (**a**–**h**) of the BC produced by *Komagataeibacter* sp. W1 grown in the media spiked with different carbon sources: (**a**) acetate, (**b**) ethanol, (**c**) fructose, (**d**) glucose, (**e**) glycerol, (**f**) lactose, (**g**) mannitol, and (**h**) sucrose. From [92], originally published under a CC-BY license.

Bacterial cellulose belongs to the most often used and investigated bacterial biopolymers [90]. Cellulose can be produced by a few bacteria, especially *Acetobacter xylinum* as well as other genera such as *Agrobacterium*, *Achromobacter*, *Rhizobium*, *Sarcina*, *Alcaligenes*, *Pseudomonas*, *Sarcina*, *Komagataeibacter,* and others [91,92,93]. The morphology and fiber diameter distribution depend, among others, on the carbon sources (Figure 6 [92]). The synthesis of bacterial cellulose ideally works with glucose as the main carbon source of the bacteria, followed by the extrusion of cellulose nanofibrils through the pores of the outer bacteria membrane and the subsequent aggregation of these nanofibrils into a web-like network [94]. Contrary to plant-based cellulose, no impurities like lignin or hemicellulose can be found in bacterial cellulose, leading to higher crystallinity and higher tensile strength [95]. Furthermore, it is relatively inert, which can be changed using conjugation with alginate, chitosan, gelatin, hyaluronic acid, or xyloglucan [96].

PHAs are another often-researched class of bacterial polymers, e.g., poly(3-hydroxybutyrate) (P3HB or PHB), poly(3-hydroxybutyrate-*co*-3-hydroxyvalerate) (PHBHV), poly(3-hydroxybutyrate-co-3-hydroxyhexanoate) (PHBHHx), and poly-4-hydroxybutyrate (P4HB) [97]. They show good biocompatibility and biodegradability and can be prepared with different side chain lengths, enabling the variation in their mechanical properties over a broad range [98]. On the other hand, bacterial polythioesters (PTEs) are not biodegradable due to the ester linkages present in their backbones but can also show thermoplastic and elastomeric properties [99].

While PHB was catalyzed in a two-stage process from *Acetobacterium woodii* which transformed carbon monoxide into formate, followed by its conversion into PHB by Methylbacterium extorquens AM1 [100], PHAs can generally be produced from beer brewery wastewater with maltose as the primary carbon source [101]. Other authors suggested food waste-streams as a possible feedstock for bacterial fermentation for PHA production [102] or other low-cost carbon sources, e.g., soy molasses, rice bran, dates [103], or vinasse-containing substrates [104].

The cationic lysine homopolymer, polylysine, contains functional carboxyl and ε-amine groups, has hydrophobic and hydrophilic properties, and is produced by *Streptomyces* bacteria [105]. 

Amongst the polyamides, poly(γ-d-glutamic acid) (γ-PGA) and poly(ε-l-lysine) (ε-PL) are segregated and form a biofilm or encapsulate the cell [106]. Regarding polyesters, polyhydroxyalkanoates (PHAs) are synthesized by many bacteria as a way to store carbon and energy [107]. Polyphosphates are used for energy and phosphate storage [108]. Amongst the proteins, there are fimbrillin, pilin, or flagellin, which can self-assemble into nanofibers or similar forms [109].

### 3.2. Typical Applications of Bacterial Biopolymers

Bacterial biopolymers can have different physical and chemical properties and are thus used in diverse applications. Bacterial cellulose is mainly used in biomedicine, as food, and in bioengineering pharmaceutics and cosmetics [110,111]. Its properties can be optimized in situ (before biosynthesis) or ex situ (after biosynthesis) for a special application [110]. It is often used for wound dressing, for which it can further be optimized by adding silver nanoparticles or ZnO to increase the antibacterial activity, hyaluronan for higher thermal stability, or agarose to improve the mechanical properties and the fluid uptake [112]. Bacterial cellulose can also be used for other diverse biomedical applications, such as medical implants [113], three-dimensional scaffolds with improved biocompatibility [114], filtration, skin and vascular grafts, or drug delivery [115]. Special bacterial polymers can even be used in cancer treatment [116], especially combined with other biopolymers and supportive substances [117].

Other potential applications of bacterial biopolymers are as substrates for Co nanoparticles used in catalytic experiments [118] and different energy applications, as discussed in the next Sections.

## 4. Algal Biopolymers for Batteries

**Figure 7:**
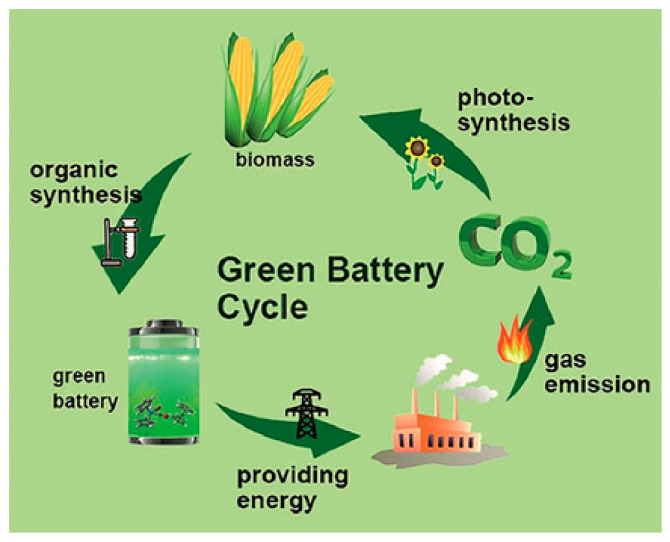
Green battery cycle. From [119], originally published under a CC-BY license.

Using biopolymers from natural sources for batteries ideally terminates the “green battery cycle”, as described by Liedel and depicted in Figure 7 [119]. In this cycle, photosynthesis of plants, e.g., algae, produces biomass, which is organically synthesized to prepare “green” batteries, which provide energy, and whose CO_2_ emission is used again for photosynthesis. 

**Figure 8:**
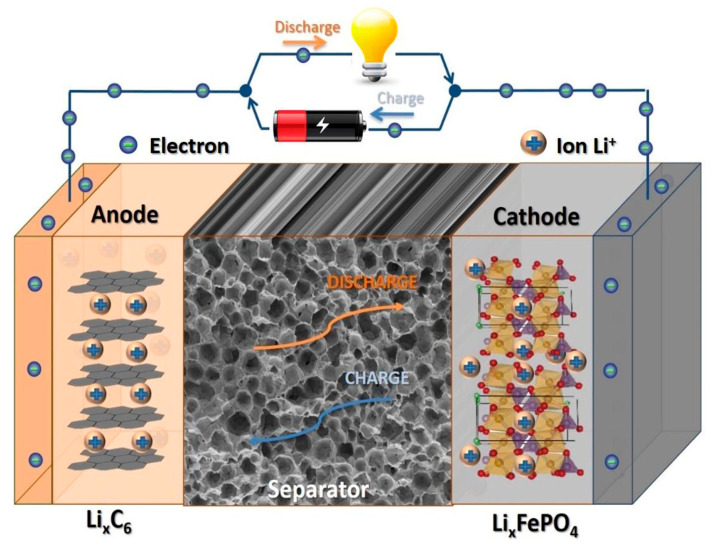
Main components of a lithium-ion battery and the charging and discharging modes. Reprinted from [120] and copyright 2019, with permission from Elsevier.

Generally, batteries consist of two electrodes (i.e., anode and cathode) and the separator between them, as shown in Figure 8 [120]. Both electrodes must have high electric conductivity and must be chemically stable. The separator allows Li ions or the corresponding charge carriers in the respective battery type to be transferred through it; thus, its porosity and permeability as well as the absorption and retention of the electrolyte are important parameters. 

Biomass from algae and other natural sources can be used for all important parts of batteries, i.e., electrodes (either in their natural form or after carbonization), binders, electrolytes, and separators [119,121]. However, mostly algal biopolymers are used as electrolytes and separators [122,123], while only few reports about algal biopolymers as electrode materials or binders can be found in the scientific literature [124,125].

### 4.1. Separator Materials

Typical materials used as separators are alginates and cellulose, while several other algae biopolymers have also been investigated as ion-conducting membranes [126]. Separators composed of sodium alginate and poly(ethylene oxide) (PEO) were used in Li metal batteries, where the sodium alginate supported the structure of the film, while PEO absorbed the liquid carbonate electrolyte and thus enabled the diffusion of Li ions [127]. Embedding nanofibers from the natural mineral attapulgite into sodium alginate, Song et al. prepared an eco-friendly porous separator with good thermal and chemical stability, which could be efficiently wetted with the liquid electrolyte in a Li-ion battery [128]. Embedding cellulose as filler in calcium alginate, Tan et al. prepared Li-ion batteries with a high capacity retention ratio of ~90%, thermal and chemical stability, and no hot melt shrinkage at high temperatures, making this battery highly safe [129]. 

Algal cellulose was received from algal waste from the food industry and blended with soy protein to prepare a sustainable separator with high ionic conductivity (5.8 mS/cm), which showed good cycling properties in Li-ion batteries [130]. Serra et al. underlined the high porosity of 80–90% of their separator membranes, where the average pore size depended on the algal cellulose content, the thermal stability of the membranes up to 150 °C, and the good mechanical properties, as compared to pure soy protein separators [130]. Algal cellulose from *Cladophora* was even used to prepare a pure paper-based battery, where the high crystallinity, porosity, and specific surface area of the algal cellulose enables using the material as a separator with good electrolyte wettability, thereby increasing capacity, stability, and safety of Li-ion batteries [131].

### 4.2. Electrolyte Materials

Most researchers concentrate on algal biopolymers as solid polymer electrolytes for batteries and other energy-storage devices, where the biopolymer forms a matrix which contains different ionic dopants, such as Li or Mg salts [132], and thus combines electrolyte and separator. Typical algal biopolymers for electrolytes in the form of membranes or hydrogels are polysaccharides, e.g., alginate or κ-carrageenan [133,134,135].

κ-carrageenan is already being used in different industries on relatively large scales; however, it is hydrophilic and has low mechanical properties, making its use as polymer electrolyte challenging [136]. Nevertheless, it is often investigated for different battery types. 

κ-carrageenan was used in Li-ion batteries by Arockia Mary et al., who added LiNO_3_ to the biopolymer to increase the ionic conductivity up to 1.9 · 10^−3^ S/cm and achieved electrochemical stability of up to 3.2 V for the optimum amount of LiNO_3_ in the membrane [137]. In the same group, LiCl salt was added to κ-carrageenan to form a membrane with ionic conductivity of 1.2 · 10^−2^ S/cm, reaching a voltage of 3.25 V in a rechargeable battery with a LiFePO_4_ cathode and an activated charcoal/graphite anode [138]. Rudati et al. added ammonium chloride to κ-carrageenan and used the resulting free-standing film as an electrolyte in an organic C/Zn battery, which reached a voltage of 2.1 V [139]. By doping κ-carrageenan with ammonium bromide, Nithya et al. prepared an electrolyte with high conductivity of 2.8 · 10^−3^ S/cm, and the battery composed of this electrolyte, a zinc/graphite anode, and a lead oxide/graphite/vanadium pentaoxide cathode showed an open-circuit voltage of 4.29 V [140]. Using a κ-carrageenan/NH_4_Cl film as an electrolyte resulted in a ionic conductivity of 3 · 10^−4^ S/cm, which was used to prepare a battery with zinc sulfate/graphite/zinc anode and vanadium pentaoxide/graphite/lead oxide cathode, which showed an open-circuit voltage of 1.74 V [141]. Especially for wearable devices, Perumal and Selvin developed flexible solid electrolytes from κ-carrageenan and NH_4_COOH, which showed a proton-conductivity of 8.5 · 10^−4^ S/cm and electrochemical stability of up to 6.3 V [142].

**Figure 9:**
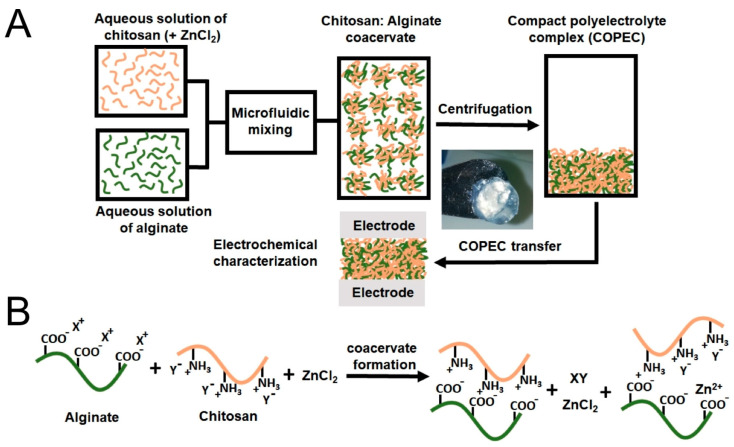
(**A**) The sequential view of the compact polyelectrolyte complexes’ preparation route; (**B**) the ideal view of the ionic interactions between the polyelectrolyte chains and the ions involved in the synthesis of the coacervate (X^+^=H^+^; Y^−^=CH_3_COO^−^). From [145], originally published under a CC-BY license.

Alginate electrolytes were prepared by Diana et al. who doped sodium alginate with sodium thiocyanate (NaSCN), resulting in an ionic conductivity of 1.2 · 10^−2^ S/cm and a measured open-circuit voltage of 2.87 V in an all-solid-state sodium-ion battery [143]. For use in magnesium-ion batteries, Tamilisai et al. embedded magnesium nitrate (Mg(NO_3_)_2_·6H_2_O) in sodium alginate, leading to a ionic conductivity of 4.6 · 10^−3^ S/cm and an open-circuit voltage of 1.93 V [144]. Blending alginate with chitosan and doping the blend with ZnCl_2_ (Figure 9), Fernández-Benito et al. prepared an aqueous zinc ion polyelectrolyte with a conductivity of approx. 10^−3^ S/cm and high electrochemical cyclability of over 7000 cycles [145].

Another algal biopolymer that has been investigated as a potential electrolyte for batteries is an agarose matrix with concentrated KOH as a liquid electrolyte for zinc–air batteries, amongst others [146]. Besides these algal biopolymers, the next Section describes bacterial biopolymers with potential use in batteries.

## 5. Bacterial Biopolymers for Batteries

Similar to algal biopolymers, many bacterial biopolymers can be used for batteries. Bacterial cellulose can be carbonized to form nano-carbon, with a very high specific surface area and used as a binder or an electrode, or it can be used without carbonization as an encapsulation material in lithium–sulfur batteries [147,148]. Another bacterial biopolymer that can be used as a binder is xanthan gum, derived from *Xanthomonas campestris*, which is water-soluble and has a high molecular weight, which was found to provide good capacity retention [149].

The carbonization of bacterial cellulose leads to interconnected porous structures and abundant oxygen-containing groups as well as the formation of oriented graphite structures [150,151,152]. Thus, such “hard” carbons are broadly investigated as anode materials for lithium-ion and sodium-ion batteries [153]. Carbonized bacterial cellulose was also used to prepare a binder-free freestanding cathode for KS (potassium sulfur) batteries by dip-coating the carbonized bacterial cellulose with sulfur/carbon disulfide and subsequently using melt diffusion at 160 °C, resulting in a capacity of 123 mAh/g after stabilization with a capacity retention of 86% after 500 cycles [154]. Other researchers reported different methods to create electrodes from bacterial cellulose, typically using carbonization or pyrolyzation, partly followed by KOH activation or similar chemical treatments [155].

**Figure 10:**
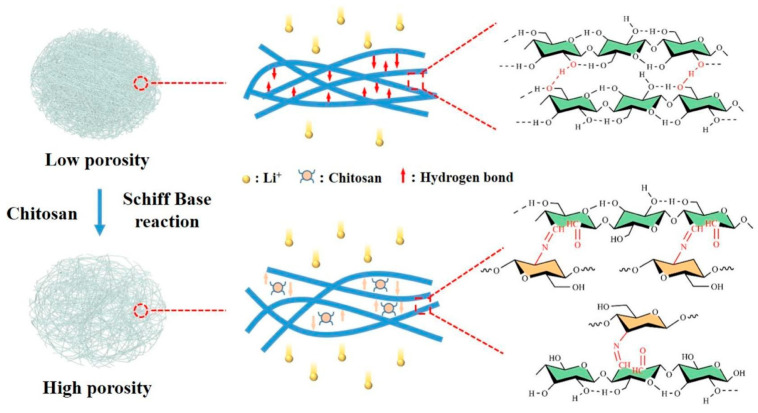
Improving pore structure and porosity of a bacterial cellulose separator by grafting chitosan on cellulose. Reprinted from [161] and copyright 2023, with permission from Elsevier.

However, bacterial cellulose is more often used as separator. Baranwal et al. prepared polydopamine(PDA)-functionalized bacterial cellulose as a separator for a lithium–sulfur battery [156,157]. Thus, polysulfide shuttling could be avoided by providing active sites where the polysulfides were trapped, prohibiting undesired migration to the anode. On the other hand, the migration of Li ions was improved by functional groups in the separator, and a uniform Li-ion flux at the Li anode was supported by the homogeneous pore distribution in the separator, and thus, a large capacity of 1450 mAh/g with a very low decrease during 650 cycles was reached [156]. Avoiding the shuttle effect of polysulfides by bacterial cellulose interlayers was also investigated in other studies in order to improve the cycle stability of different batteries [158]. Combining bacterial cellulose with Al_2_O_3_, Ulfa et al. achieved a higher crystallinity than that of pure bacterial cellulose as well as increased porosity, electrolyte absorption, and conductivity, suggesting the use of this composite as a battery separator [159]. Heydorn et al. investigated bacterial cellulose separators especially for nickel–zinc batteries and found high hydroxide and zincate ion diffusion as well as high electrolyte uptake for a porous separator and better zincate shielding for a denser separator, while combining both resulted in slower cell aging and less ZnO in the pores of the separator [160]. For Li-ion batteries, Chen et al. suggested a bacterial cellulose/chitosan separator whose pore size could be tailored, as depicted in Figure 10 [161]. They found improved pore structure and porosity as well as dispersion uniformity due to chitosan grafting. The separator showed electrolyte absorption of more than 300%, high ionic conductivity, and good interface compatibility. The battery prepared with this separator had a high capacity retention of 90% after 100 cycles and a specific capacity of 150 mAh/g [161]. Huang et al. used zeolitic imidazolate framework-67 (ZIF-67) instead to form a composite separator with bacterial cellulose in order to improve the pore structure and increase the electrolyte retention capability as well as to strongly increase the ionic conductivity, leading to capacity retention of 91.4% after 100 cycles and a large capacity of 156 mAh/g [162]. Combining bacterial cellulose with polyether block amide, Ajkidkarn and Manuspiya prepared a highly porous membrane with high wettability and electrolyte uptake as well as good ionic conductivity, thermal stability, and mechanical properties, which could be used as a separator for Li-ion batteries [163].

Bacterial cellulose and other bacterial biopolymers can also be used as electrolytes in different batteries. Shi et al. used a gel electrolyte from bacterial cellulose with LiI as the redox mediator for Li-O_2_ batteries that reduced the I^3−^ ion shuttle effect and thus led to good cycling performance without self-discharge [164]. Bacterial cellulose can also be blended with poly(vinyl alcohol) (PVA) to prepare flexible, conductive, mechanically strong solid electrolyte materials for rechargeable zinc–air batteries [165].

Besides bacterial cellulose, gellan gum is also often used as an electrolyte in batteries. Buvaneshwari et al. prepared gellan gum electrolytes with Mg(ClO_4_)_2_ salts as magnesium ion-conducting solid electrolyte and found a ionic conductivity of up to 10^−2^ S/cm and an open-circuit voltage of the magnesium battery prepared with this electrolyte of 2.52 V [166]. Combined with sodium perchlorate (NaClO_4_), gellan gum was also used as a solid electrolyte for solid-state sodium-ion batteries, where the addition of the salt increased the ionic conductivity from 3.87 · 10^−6^ S/cm for pure gellan gum to 4.85 · 10^−3^ S/cm, and an open circuit voltage of 2.99 V was found for the sodium ion battery [167]. By adding ammonium thiocyanate (NH_4_SCN) salt to gellan gum, the same group produced an electrolyte with proton conductivity of 1.41 · 10^−2^ S/cm and a corresponding proton battery with open circuit voltage of 1.62 V [168].

After describing the large potential of algal and bacterial biopolymers for applications in batteries, their potential use as biofuel will be discussed in the next Sections.

## 6. Algal Biopolymers for Biofuel

Algal biomass can be used to produce liquid, gaseous, or solid biofuels, such as bioethanol, bio-methane, biodiesel, bio-hydrogen, or bio-gas [169]. Bioethanol is produced with the fermentation or gasification of algal polysaccharides, mostly from brown algae, which have high carbohydrate content, but also from green and read algae [170]. For bio-methane, the anaerobic digestion of algae can be used [171], while biodiesel is derived from oils or lipids produced by microalgae [172]. Bio-hydrogen can be produced by green algae under anaerobic conditions [173], while biogas results from the anaerobic digestion of microalgae [174]. For detailed overviews, please see reviews [175,176,177].

While biomass from microalgae allows producing biopolymers, biofertilizers, and biofuels at the same time, the extraction of these components from the microalgae is more complicated and thus less economical than from other sources [178,179]. This makes producing biofuel from algal biomass commercially challenging and suggests combining biofuel production with the extraction of high-value components from algae, such as therapeutics, nutraceuticals, and cosmetics [41,180]. On the other hand, waste-streams can be better managed if they are used as feedstock for algal growth, thereby using a waste-as-a-value approach that reduces the overall costs for the received algal products [181,182]. Furthermore, microalgal biomass can be grown on non-arable land and shows higher productivity of usable biomass than terrestrial plants, making algae advantageous for biofuel production [182].

**Figure 11:**
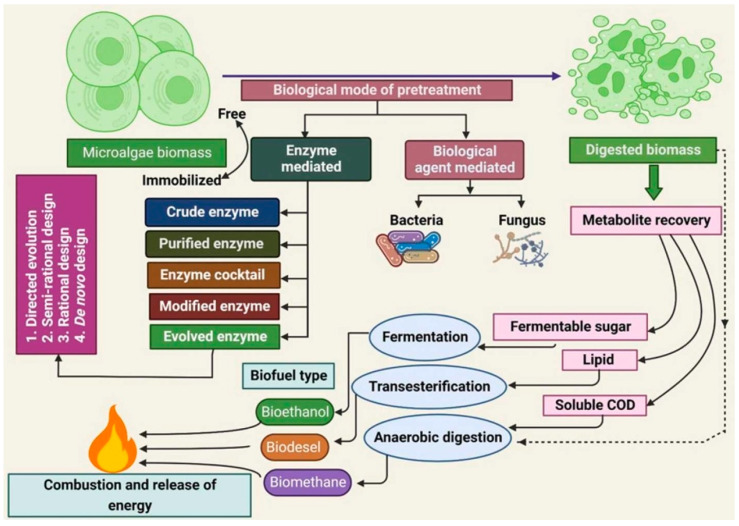
Biological pretreatment methods to obtain biofuel from algal biomass feedstock. Reprinted from [188] and copyright 2023, with permission from Elsevier.

Generally, bioenergy feedstock should contain a large amount of biopolymers, making especially green and blue microalgae interesting for biofuel generation [183,184]. Cellulose belongs to the class of biopolymers that can be used for biofuel production by simultaneous saccharification and either fermentation or co-fermentation after a suitable pretreatment of the cellulose feedstock [185]. Pretreatment is necessary to disintegrate the cell walls of the algal biomass in order to release the intracellular biopolymers [186,187]. Biological pretreatment can occur in different ways, as depicted in Figure 11, and it is either enzyme-mediated or biological agent-mediated [188].

**Figure 12:**
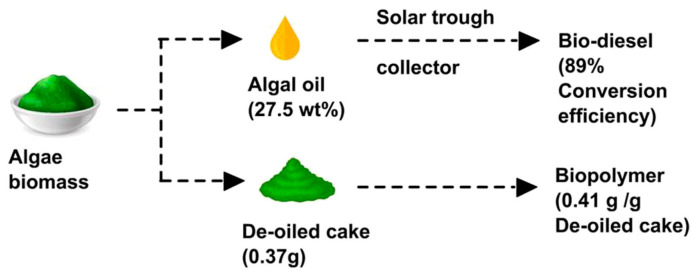
Production of algal oil for biodiesel and the biopolymer PHB from the de-oiled cake at the same time. Reprinted from [189] and copyright 2022, with permission from Elsevier.

Biofuel production from algae is thus ideally combined with either wastewater cleaning or the production of high-value products. Arun et al. suggested a biorefinery concept in which algal oil was extracted from *C. vulgaris* biomass in order to produce biodiesel as well as the biopolymer PHB from the de-oiled cake, as depicted in Figure 12 [189]. The combined production of biofuel and PHB from *Chlorella pyrenoidosa* was described by Das et al. [190]. Different pretreatments, such as acidic, enzymatic, and microwave laser-hydrogen peroxide-Fe-nanoparticle pretreatment, were tested by AlMomani et al. with respect to their effect on the production of bioethanol and biopolymer from algal biomass [191]. On the other hand, Kumar et al. concentrated on the de-oiled algal biomass pretreated using a hybrid physicochemical/enzymatic method and found that it could be used as a feedstock for bioethanol as well as biopolymer (PHB) production; thus, the whole de-oiled algal biomass could be used without producing any waste [192]. Vickram et al. also described algal biomass and as a feedstock for biofuel production and the biopolymer PHA, but also the acid hydrolysis of PHA to receive biofuels from methyl esters of hydroxyalkanoates (HAME) and hydroxybutyrate (HBME) [193].

It is evident from these examples that the production of biofuels from algal biomass, often combined with the extraction of polymers, has several advantages but also economic challenges. The next Section investigates the opportunities and challenges for the biofuel production from bacterial biopolymers.

## 7. Bacterial Biopolymers for Biofuel

**Figure 13:**
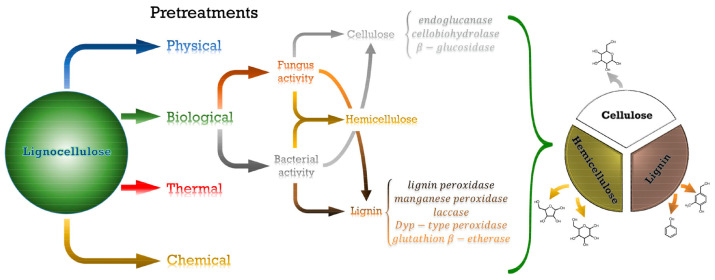
The main pretreatments of lignocellulose and obtained products, with special attention provided to the enzymes involved in both the fungal and bacterial biological processes of cellulose and lignin degradation. Reprinted from [194], originally published under a CC-BY license.

One of the possibilities to prepare biofuel from bacterial biopolymers is provided by the use of lignocellulosic material, which is pretreated with bacteria, leading to the biodegradation of cellulose, hemicellulose, and lignin in different amounts, depending on the chosen bacteria (Figure 13) [194]. Bacterial pretreatment can be finished within some hours to days, while pretreatment with white-rot fungi usually takes from weeks to months; nevertheless, it is often used due to its high efficiency [195]. Generally, the production of biofuel from lignocellulosic feedstock is reasonable since this material is highly abundant and can be found in large amounts in woody and non-woody plants; however, to make the process economical, biofuel production is often combined with the production of biopolymers, industrial biocatalysts, and other high-value products [196]. Furthermore, it is possible to use agro-industrial waste as feedstock, thereby achieving an environmentally friendly bioconversion biofuel and other into viable bioproducts [197,198]. Among the often-used bacteria, *Pseudomonas putida, Rhodococcus pyridinivorans* CCZU-B16, *Arthrobacter* sp. C2, and *Caldicellulosiruptor kronotskyensis* are used for the depolymerization of lignin [199].

Besides lignocellulosic material, several other biopolymers and other materials can be used for biofuel production by bacteria. Banu et al. investigated the possibility to use waste-activated sludge that can contain harmful pathogens and other problematic organic and inorganic substances for bioenergy production [200]. For this purpose, they pretreated the sludge using bacterial disintegration, supported by a TiO_2_-embedded chitosan thin film that increased this effect by an increased hydrolytic activity [200]. PHAs, which can be produced by microalgae as well as by diverse bacteria, are often used as a base for the production of HAME biofuels, as already discussed in Section 6 [20,201]. De Paula et al. showed PHA production from crude glycerol as the only carbon source by the newly found bacterium *Burkholderia glumae* MA13 [202]. On the other hand, *Rhodopseudomonas palustris* can not only degrade lignocellulosic biomass hydrolysates but also assimilate short-chain organic acids and crude glycerol from agricultural as well as industrial wastewater [203]. Glucose was used as feedstock for *Clostridium acetobutylicum* NCIM 2337 to produce biohydrogen, biobutanol, and an undefined biopolymer [204]. Special bacteria, called methanotrophs, can metabolize methane to produce methanol as well as exo-polysaccharides [205]. Similarly, *Ralstonia eutropha* and similar bacteria can produce biofuels from CO_2_ and H_2_ using fermentation [206].

## 8. Discussion

### 8.1. Advantages and Disadvantages of Algae-Based and Bacterial Biopolymers

As evident from the previous Sections, algae-based biopolymers and bacterial biopolymers have both emerged as promising candidates for applications in batteries and biofuels, with unique advantages and different challenges. 

Algae-based biopolymers are potentially carbon neutral as they are derived from renewable and biodegradable algae sources. They possess great potential for battery applications due to their high surface area, porosity, and conductivity, which in turn boost electrode performance and stability [207]. Additionally, algae-based biopolymers reduce environmental load compared to conventional electrode materials due to their sustainable nature. However, challenges such as scalability and cost-effectiveness still need to be addressed to make them commercially viable for large-scale battery production [208]. 

Bacterial biopolymers such as PHAs have exhibited their potential in biofuel applications due to their biodegradability, high energy content, and compatibility with existing fuel infrastructure [209]. PHAs are produced from various renewable feedstocks, including agricultural and industrial waste, offering a sustainable carbon-deficient solution for biofuel production. However, slow growth rates, slow bacterial polymer accumulation, and the optimization of fermentation processes deter their widespread implementation and competitiveness with conventional fuels [210].

Despite these challenges, both algae-based and bacterial biopolymers hold significant potential for advancing sustainable energy technologies. Algae-based biopolymers and bacterial biopolymers both show potential for applications in batteries and biofuel production. Algae-based biopolymers offer advantages such as their high surface area and porosity, which can enhance electrode performance and electrolyte absorption, leading to improved battery efficiency in battery applications [211]. The abundant availability of algae as a renewable resource contributes to their appeal for sustainable energy applications. However, algae-based biopolymers face challenges related to scale-up and cost-effectiveness in battery production due to the complexity of cultivation, contamination, and bioprocessing [212]. On the other hand, bacterial biopolymers offer distinct advantages for biofuel applications due to their compatibility with the existing infrastructure for biofuel production [213]. Bacterial biopolymers can be derived from various wastewater sources. Bacterial biopolymers such as PHAs can be utilized as precursors for biofuels, offering a renewable and biodegradable alternative to fossil fuels. Low energy density and poor mechanical properties deter their widespread implementation in high-performance biofuel applications [214]. Algae-based biopolymers are more suitable for battery applications with their unique properties, whereas bacterial biopolymers are more suitable for biofuel production, each presenting distinct advantages and challenges.

### 8.2. Environmental Suitability of Algae-Based and Bacterial Biopolymers

The sustainability and ecological impact of using algae-based and bacterial biopolymers are crucial considerations in evaluating their environmental suitability. Life cycle assessments (LCAs) provide valuable insights into the carbon footprint of the biopolymers across their entire life cycle, i.e., from production to disposal. Algae-based biopolymers are promising candidates due to their renewable, biodegradable nature and their carbon sequestration potential during cultivation. 

Microalgae utilize carbon dioxide during photosynthesis and mitigate greenhouse gas emissions [215]. Algal cultivation can utilize non-arable land and wastewater, thus minimizing competition with food production [216]. However, LCAs have exposed problems such as high energy consumption and high water usage in algal cultivation and processing [217]. 

On the other hand, bacterial biopolymers like PHAs offer sustainability advantages through their production from renewable resources and biodegradability. PHAs are synthesized by bacteria that utilize various carbon sources, including agricultural waste and industrial by-products, reducing our dependence on fossil fuels [218]. These resources minimize environmental impacts linked to conventional plastics derived from petroleum. PHAs are biodegradable under natural conditions, potentially reducing plastic pollution and ecosystem damage [219]. However, LCAs have revealed energy-intensive fermentation processes and limited end-of-life options for PHAs, such as industrial composting facilities, which may not be commonly accessible [220].

Despite these challenges, both algae-based and bacterial biopolymers provide environmental benefits than conventional plastics. Research in the optimization of production processes and sustainable end-of-life solutions can enhance their ecological performance.

## 9. Conclusions

It is evident from this review that there are numerous possibilities for using algae or bacteria for the production of biopolymers that can be utilized in different parts of batteries or can be used as the base for biofuels, as well as for the simultaneous production of biopolymers and biofuel. Biological production methods are advantageous due to their increased sustainability. On the other hand, several problems still reduce the usability of algae-based and bacterial biopolymers. Low scalability and cost-effectiveness impede the broader use of algal biopolymers, while bacterial polymers exhibit slow growth rates, slow bacterial polymer accumulation, and non-optimal fermentation processes. On the application side, bacterial biopolymers often show low energy density in biofuels and low mechanical properties. These disadvantages can be overcome by optimizing production and harvesting methods as well as blending algae-based and bacterial biopolymers with other sustainable materials to improve their performance in battery and biofuel applications. 

To further make the production methods economically feasible, two possibilities for addressing these economic challenges have been suggested and investigated: the co-production of biofuel with high-value products and the transformation of the production process to metabolize waste, CO_2_, or other undesired resources within a waste-to-wealth process.

## Data Availability

No data were produced in this review.
